# Integrating CT Volume and Plasma *p* ‐Tau217 for Amyloid Risk Stratification in Early Stage Alzheimer’s Disease

**DOI:** 10.1002/alz70861_108394

**Published:** 2025-12-23

**Authors:** Sohyun YIM, Seongbeom Park, Kichang Kwak, Sang Won Seo

**Affiliations:** ^1^ Samsung Medical Center, Gangnam‐gu, Seoul Korea, Republic of (South); ^2^ BeauBrain Healthcare Inc, Seoul, Seoul Korea, Republic of (South); ^3^ SAIHST, Sungkyunkwan University, Seoul Korea, Republic of (South); ^4^ Sungkyunkwan University, Suwon Korea, Republic of (South); ^5^ Samsung Medical Center, Sungkyunkwan University School of Medicine, Seoul Korea, Republic of (South)

## Abstract

**Background:**

With the emergence of Aβ‐targeted therapies, detecting amyloid‐β (Aβ) deposition in early stage Alzheimer’s disease (AD) is essential for timely management. Although Aβ positron emission tomography (PET) and cerebrospinal fluid (CSF) testing are reliable, their limited accessibility restricts routine uses. In response, we developed a two‐stage diagnostic workflow integrating computed tomography (CT)‐based brain atrophy pattern and plasma *p* ‐Tau217 testing to predict Aβ PET positivity. We further compared this CT‐based workflow with a previously validated MRI‐based workflow.

**Method:**

This cohort study included participants diagnosed with mild cognitive impairment (MCI) or early dementia of Alzheimer’s type (DAT) from the K‐ROAD cohort (*n* =617). In the first stage, a random forest classifier was constructed to predict Aβ PET positivity using CT‐derived brain atrophy patterns and *APOE ε4* status. Based on the prediction from this classifier, participants were stratified into low‐, intermediate‐, and high‐risk groups for Aβ PET positivity. In the second stage, plasma *p* ‐Tau217 testing was applied exclusively among individuals classified within the intermediate‐risk group. The accuracy of the data was evaluated using positive predictive value (PPV), and negative predictive value (NPV).

**Result:**

In the first stage of the workflow, using the 95% sensitivity/specificity strategy, the NPV in the low‐risk group was 95.8% (92.9–98.7%) and the PPV in the high‐risk group was 98.4% (96.9‐99.9%). 28.2% of participants were classified into the intermediate‐risk group. In the second stage, plasma *p* ‐Tau217 testing for intermediate‐risk participants achieved a PPV of 96.9% (93.9‐100.0%) and a NPV of 74.4% (61.4‐87.4%). The overall accuracy of the workflow was 95.6% (94.0‐97.2%).

**Conclusion:**

This study demonstrates that the CT‐based two‐stage diagnostic workflow can accurately predict Aβ PET positivity in early‐stage AD. This approach reduces uncertainty in the intermediate‐risk group and minimizes unnecessary testing. It provides a cost‐effective and scalable alternative to PET and CSF testing, optimizing healthcare resource allocation in clinical practice.

